# Pleiotropic Effects of Resveratrol on Aging-Related Cardiovascular Diseases—What Can We Learn from Research in Dogs?

**DOI:** 10.3390/cells13201732

**Published:** 2024-10-18

**Authors:** Arkadiusz Grzeczka, Szymon Graczyk, Pawel Kordowitzki

**Affiliations:** Department for Basic and Preclinical Sciences, Faculty of Biological and Veterinary Sciences, Nicolaus Copernicus University, 87-100 Toruń, Poland; grzeczka@umk.pl (A.G.);

**Keywords:** trans-3,5,4′-trihydroxystilbene, resveratrol, heart, dog, antioxidant, cardiovascular

## Abstract

Resveratrol (RES) is a polyphenol with natural anti-inflammatory and antioxidant properties. It is found in abundance in plants, i.e., grapes and mulberry fruit. In addition, synthetic forms of RES exist. Since the discovery of its specific biological properties, RES has emerged as a candidate substance not only with modeling effects on the immune response but also as an important factor in preventing the onset and progression of cardiovascular disease (CVD). Previous research provided strong evidence of the effects of RES on platelets, mitochondria, cardiomyocytes, and vascular endothelial function. In addition, RES positively affects the coagulation system and vasodilatory function and improves blood flow. Not only in humans but also in veterinary medicine, cardiovascular diseases have one of the highest incidence rates. Canine and human species co-evolved and share recent evolutionary selection processes, and interestingly, numerous pathologies of companion dogs have a human counterpart. Knowledge of the impact of RES on the cardiovascular system of dogs is becoming clearer in the literature. Dogs have long been recognized as valuable animal models for the study of various human diseases as they share many physiological and genetic similarities with humans. In this review, we aim to shed light on the pleiotropic effects of resveratrol on cardiovascular health in dogs as a translational model for human cardiovascular diseases.

## 1. Introduction

Dogs have long been recognized as valuable animal models for the study of various human diseases as they share many physiological and genetic similarities with humans. One area of particular interest is the use of dogs as a translational model for human cardiovascular diseases. Therefore, the canine species has attracted researchers to investigate human diseases in companion dogs. One key advantage of using dogs as a translational model is their ability to naturally develop cardiovascular diseases, such as heart failure, mitral valve disease, and arrhythmias, which closely mimic the progression and characteristics observed in humans [Box 1]. This is particularly important as it allows us to study the pathogenesis, progression, and potential treatments for these conditions in a more clinically relevant setting, as opposed to relying solely on laboratory-induced disease models. Several breeds of domestic dogs represent a unique animal model since companion dogs share the same environment with people and influence the quality of life for millions of us. Resveratrol (RES) (trans-resveratrol; trans-3,5,4′-trihydroxystilbene), a polyphenol phytoalexin, is a non-flavonoid polyphenol. RES has two isomers, cis and trans, the latter of which is the best studied and responsible for biological activity. It is a naturally occurring antioxidant that plants use as one form of protection against fungi [1]. It is found in the highest concentrations in mulberry and bilberry fruit, but the most widely commented upon source of RES is red grapes and the food and alcohol products derived from them [2]. It has antioxidant and anti-inflammatory properties. Noteworthily, its cardioprotective activity is related to the ability of RES to regulate superoxide dismutase (SOD) activity, the SIRT1/AMPK pathway, the arachidonic acid (AA) pathway, or the activity of the nuclear transcription factor NF kappa B (NF-κB), all of which play important roles in inflammatory and immune processes [2,3]. RES-containing foods are widely appreciated by scientists, nutritionists, and physicians and are used in different fields of human medicine [4]. Patients suffering from obesity, diabetes, fungal infections, neurodegenerative diseases such as Alzheimer’s, fertility disorders, age-related diseases, and even cancer benefit from providing RES via food, supplementation, or increasing RES levels with other foods [5]. The anti-aging mechanisms of RES, consisting mainly of alleviating oxidative stress, moderating the inflammatory response, or improving mitochondrial function, lead us to believe that RES is also potentially a protective compound that, when taken chronically—including in health—will slow down the aging process and protect the body against the development of diseases, especially those that occur more frequently with age [6,7].

Particular attention is paid to the cardioprotective effects of RES. This is justified by the fact that the main factors for the onset and progression of CVD are inflammation, age, and lifestyle, as well as a genetic component [8]. The conjecture began with the observation that populations of people who consumed greater amounts of red wine (and therefore greater amounts of RES), such as the French, were less likely to die of coronary heart disease—an effect dubbed the “French paradox” [9,10]. In particular, RES is attributed with antiatherosclerosis, antihypertensive, protective effects against stroke, myocardial ischemia, heart failure, protective effects on the vascular endothelium, and anticoagulant effects [11,12]. Based on the results demonstrating RES as a supplement that alleviates left atrial remodeling, improves left ventricular diastolic function, and nullifies cardiac fibrosis in hypertensive patients, it is conceivable that in the future, it may become part of adjuvant therapy or become an adjunct to conventional treatments [13]. The question among veterinarians is whether RES will have a similar cardioprotective effect and be useful in veterinary patients. This question is legitimate because of the different nature of the medical care offered to animals and also because of interspecies physiological differences. It has been indicated that the effects of RES on canine health may be positive [14,15,16]. However, some cardiovascular disorders common in humans, i.e., stroke secondary to atrial fibrillation or atherosclerosis, against which RES has a protective effect, do not occur in animals, which may reduce its usefulness in veterinary medicine. Atrial thrombosis, which is a cause of strokes, is incidental, and only a few papers provide evidence of its possibility in dogs [17]. As well, atherosclerosis in dogs or cats is difficult to induce, even experimentally. Atherosclerotic lesions obtained in dogs have a similar location to humans (abdominal aorta and iliac arteries) [18], and histological changes are characteristic plaque lesions in the middle layer of the arteries [19]. However, research on dogs is limited for ethical and financial reasons (especially attempts at genetic induction of atherosclerosis) [20].

In addition, dogs and cats (the main patients dealt with by veterinary cardiologists) live much shorter lives than humans, so the inhibitory effect on aging may be less pronounced or even absent. Veterinary patients, on the other hand, have a predisposition to pulmonary, aortic, distal arterial, and venous thromboembolic diseases [21], degenerative diseases of the atrioventricular valves, heart failure, cardiomyopathy, or some arrhythmias [22]. Furthermore, as in humans, inflammation is recognized as a risk factor for the onset and progression of CVD in dogs. In addition, it is worth mentioning that the first study that reassured researchers that compounds in grapes/red wine may be related to the cardioprotective effects of these products was carried out specifically on dogs, in which RES improved flow in experimentally constricted coronary vessels [23].

Because of species-specific low b-glucuronidase activity, cats do not metabolize, or only slightly metabolize, trans-RES, and studies have indicated significant urinary complications [24]. Therefore, this work aims to summarize the current knowledge on the possible health benefits of RES supplementation in the treatment of CVD in dogs. We focus on aspects of anti-inflammatory and antioxidant effects as well as possible cardioprotective effects of RES.

Box 1 **The dog is a model of cardiovascular disease developing spontaneously.**

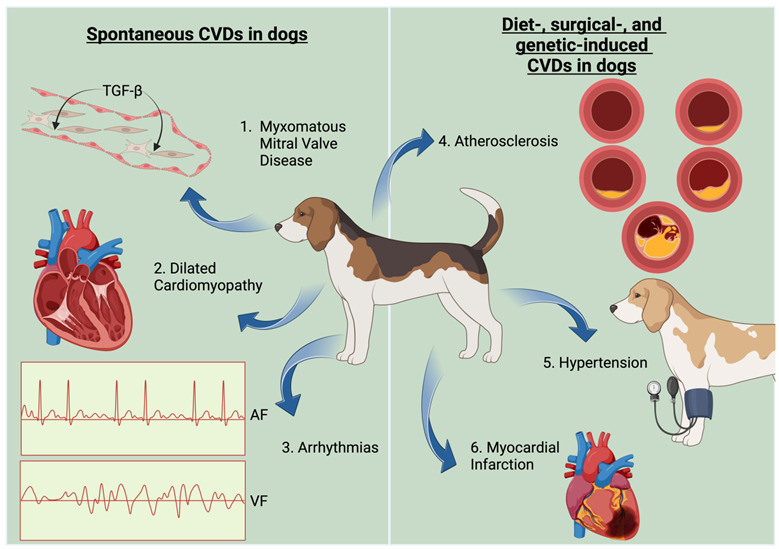

The spontaneous development of cardiovascular diseases (CVDs), which in its pathophysiology corresponds to diseases that occur in humans, creates a unique opportunity to study these diseases. Furthermore, the dog, sharing its living environment with humans, can be subjected to similar environmental factors that have a real impact on cardiovascular health (a significant advantage over laboratory animals). The domestic dog is also an established model of aging and shares key similarities with humans, including in the course and regulation of cellular senescence and systemic aging processes. Consequently, there is a strong similarity between the two species in the development of age-related CVD. Myxomatous mitral valve disease (1) and cardiac arrhythmias (3) carry distinct changes that suggest their association with the molecular mechanisms of aging, in humans as well as in dogs [25,26,27]. This phenomenon makes it possible to look deeply into the pathophysiology of diseases and compare them with healthy individuals (without the need to use genetically controlled rodents, in which inducing a multifactorial age-related disease such as MMVD realistically seems marginally effective). In addition, several breeds, mainly boxers, are predisposed to develop dilated cardiomyopathy (2), and ventricular arrhythmias (3) [28,29]. Furthermore, the diagnosis and treatment of canine heart disease represent one of the most rapidly developing branches of veterinary medicine, often assimilating implementable methods from human medicine [30]. Because of its gentle disposition and human-like size, the dog is also a useful experimental model. Using genetic or dietary modifications, vascular lesions similar to human atherosclerosis can be induced [20]. In contrast, the size of the animal allows for surgical procedures that induce hypertension or myocardial infarction to be performed [31,32,33].

## 2. Metabolism of Resveratrol in Dogs

RES is available to humans in a variety of natural and synthetic sources, but in dogs, the consumption of some fruits can lead to dramatic renal tubular degeneration [34,35,36,37]. Indeed, it appears that grapes, which are relatively rich in RES, also contain high concentrations of dog-specific nephrotoxin and tartaric acid [38]. Incidental administration of products containing tartaric acid leads to adverse health effects, including vomiting and renal failure [37]. An explanation for the different sensitivity of interspecies to tartaric acid is the difference in the expression of organic anion transporter 4 (OAT 4) in the renal tubular wall, which is responsible for the efficient excretion of tartaric acid [38]. OAT 4 shows significant species differences by its place of expression. In humans, its activity was detected along the entire tubule [39], while in mice [40] and rats, [41] the place of expression was more condensed. In dogs, the tissue distribution of OAT 4 showed the highest expression in epididymides and weak expression in the kidneys, and in primates (including humans), the highest expression was in the kidneys [42]. Decreased expression of OAT 4 may explain the higher sensitivity of the dog.

The absence of disorders of the excretory system in the case of RES-rich grape extracts may be explained by their low tartaric acid content (since its toxicity is dose- and length-dependent) [43,44]. Because of the still scarce data on natural sources of RES available to dogs, it is difficult to approve natural feed additives for use in dogs [45]. However, the data on the absence of mortality and clinical signs of toxicity in both sexes after daily (for 13 weeks) oral administration of RES to beagle dogs for 91 days at doses of 4000, 12,000, or 24,000 mg per day are encouraging [46]. Johnson et al. 2011, also showed a no-observed-effect level (NOEL) of 600 mg/kg/day. At higher doses, there was a decrease in body weight observed in both sexes [46]. In addition, RES suspended in 10 g/L carboxymethylcellulose (CMC) and administered orally at a dose of 100 mg/kg body weight had a beneficial effect on renal parameters, reducing blood urea nitrogen (BUN) and creatinine levels, compared with the control group [16]. RES, despite its significant biological effects, unfortunately, has a low bioavailability, which in different species is around 2% [47,48,49], therefore, medicine seeks to improve the transport, action, and bioavailability of RES [50,51]. RES is rapidly metabolized, as it reaches its maximum concentration after only 1.5–2 h, which is not necessarily an advantage for a supplement used over a longer period [52,53]. In dogs, peak concentrations of RES metabolites were reached after just 30 min [43]. In one study, a second peak concentration in some dogs was detected in plasma about 8 h after oral administration, which the authors explained by enterohepatic recirculation [48]. RES is absorbed by passive diffusion or transport via transport proteins across the cell membrane of enterocytes. RES then undergoes glucuronidation and sulfation processes, whereby 95% of total RES is metabolized to RES-3-O-glucuronide, -4′-O-glucuronide, -3-O-sulphate, -4′-O-sulphate, and RES–sulfate glucuronide [54].

Glucuronidation (UGT) in liver microsomes occurs similarly in dogs and humans. A comparison of paracetamol-induced UGT enzyme activity in dogs, humans, cats, and rats was performed, which indicated a baseline 6-fold lower activity UGT in cats compared with the rest of the species [55]. Similarly, when RES metabolism in cats was analyzed, no glucuronidation products of resveratrol were detected [24]. In dogs, glucuronidation of applied RES occurs with approximately 65% efficiency [56]. However, increasing the supply of RES significantly reduces its efficiency, suggesting that RES metabolism in the short term may be limited. So far, the preferential length of time between the first and second administration of RES to dogs has not been determined, and this is likely important for the effect of RES, as activity is largely dependent on the concentration of RES [43]. The concentration of metabolites increases in proportion to the dose of the parent substance and reaches higher values than the substance administered [43,46]. However, the exact process by which RES exerts its biological effects is not known. It has been suggested that circulating metabolites are responsible for the biological effects [57] or are inactive reservoir elements for RES [58]. Because of the possibility of RES accumulation in tissues [52], it has been suggested that its intracellular levels may be more relevant to the therapeutic effect than serum levels [46,59]. An alternative to the use of pure RES is piceid (trans-resveratrol-3-O-glucoside), a stilbenoid glucoside—the main RES derivative in grape juices. When administered orally, it undergoes coupling with glucuronic acid and sulfate [60,61]. RES in dogs is similarly excreted in urine and feces as in humans [46,52,60]. The relatively similar pharmacokinetics of RES, as well as sulfate and glucuronide metabolites in humans and dogs, suggest that it may have similar cardiovascular effects.

## 3. The Immune System and Resveratrol

The primary action of RES is to influence the response of the immune system. Inflammation develops as a result of the body’s physiological response to environmental influences. However, it also plays a role in the development of CVD, sometimes being a pillar of the pathophysiology of the disease. Therefore, it has been identified as one of the main risk factors for the development of CVD in dogs [62,63]. The anti-inflammatory and antioxidant effects of RES have been repeatedly demonstrated in vitro [64], in in vivo experiments, and in vivo among human patients [11,65]. However, most experimental studies have been conducted in mice or rats, which is not always directly reflective of other species. On the other hand, clinical studies using RES in canine patients are still few, and the dose producing the desired effect is not defined. Therefore, the exact effect of RES on inflammation in dogs is debatable. It is, therefore, not surprising that there is increasing interest in the levels of inflammatory mediators in cardiovascular disease and the possibilities to modify them.

The transformation of the positive effect of inflammation into an element of disease progression occurs as a result of an imbalance in its generation and reduction. The imbalance is most often related to the exhaustion of the compensatory capacity of the disease or is due to a hypersensitive response of the immune system. The cumulative effects of inflammatory cell activity are also associated with aging. Deteriorating vital functions results in a steady increase in cytokines as well as oxidative deterioration. Inflammaging, a term describing the age-dependence of inflammation severity, has been around for about 20 years and has been linked to the development of CVD in both humans and dogs [66,67,68,69]. Inflammatory markers are released from failing myocardial cells, endothelial cells, leukocytes, and platelets [70]. The literature indicates increased levels of interleukins, CRP, TNF-a lymphocytes, and neutrophils in circulating blood in canine CVD [63,71,72,73]. Higher levels of Il-1b, among others, have been indicated in dogs with congestive heart failure [73]. Pro-inflammatory cytokines, especially TNF-α, IL-1, and IL-6, exacerbate hemodynamic abnormalities and have adverse effects on the heart [74]. Inflammatory factors that are also “the hallmarks of aging” in dogs include TNF-a, IL-1b, and IL-6 [69]. Consistent with this, young dogs have lower IL-6 concentrations than adult and geriatric dogs. It is, therefore, not surprising to conclude that young dogs are less likely to develop CVD [68]. The development of heart disease is also associated with the impairment of energy pathways, including SIRT/AMPK and mitochondrial dysfunction, which is also associated with aging. Consequently, there is an impairment of antioxidant mechanisms, accumulation of reactive oxygen species (ROS), and a shift from oxidative eustress to oxidative distress [75,76]. ROS are molecules that readily interact, whose activity can be measured in dogs by, among others, malondialdehyde (associated with lipid peroxidation) and 8-hydroxy-deoxyguanosine (associated with oxidative DNA damage) [77]. Reducing inflammation improves cardiovascular parameters, making antioxidants an interesting adjunctive therapy [78]. Therefore, in atrioventricular degenerative valve disease, antioxidants are listed among the key dietary components, and oxidative status is one of the prognostic tools in congestive heart failure [77,79,80].

### 3.1. Anti-Inflammatory and Antioxidant Effects

**Figure 1:**
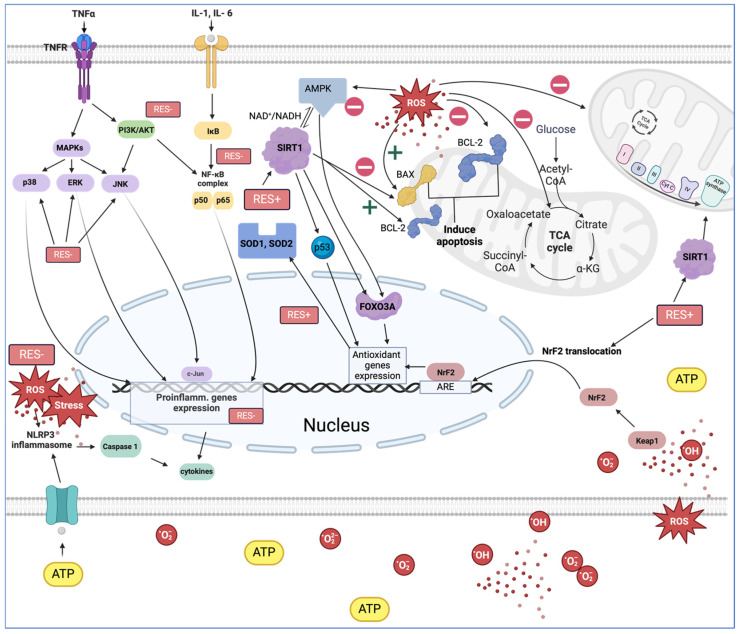
Molecular targets of resveratrol. Resveratrol exerts a negative effect (RES-) on cellular pro-oxidation mechanisms (NF-κB, MAPKs, NLRP3), resulting in reduced cytokine production. Resveratrol also exerts inhibitory effects on key pathways of the cellular inflammatory and oxidative response (PI3K/AKT). At the same time, it supports the action of NrF2 and FOXO3A in increasing the intensity (RES+) of the expression of antioxidant enzymes (SOD1, SOD2). Resveratrol, as an activator of SIRT1, indirectly influences mitochondrial metabolic efficiency by regulating the activity of the respiratory chain. Furthermore, it nullifies the negative effects of ROS on mitochondria.

RES exhibits simultaneous ROS scavenging activity and is able to interact with several important inflammatory signaling pathways and endogenous antioxidant and prooxidant processes [81,82]. The interaction between RES and the free radical response follows the reaction shown in Figure 1 and leads to a reduction in hydroxyl free radicals in vitro in canine cell lines [83]. However, we now know that the other mechanisms of RES activity provide most of its biological effects. A key finding was the demonstration of a link between RES and SIRT1 activation [84]. The sirtuin family is a group of seven nicotinamide adenine dinucleotide (NAD+)-dependent histone deacetylases whose members regulate numerous processes responsible for inflammation, oxidative status, cell cycle, energy balance, proliferation or lipid, and sugar metabolism [5,85]. Their biological activity is concentration-dependent, and, as has been shown in both small- and large-breed dogs, SIRT1 levels decrease with age [86]. A sustained decrease in SIRT1 levels can lead to impairment of these functions. In humans, SIRT1 regulates inflammation mainly via inhibition of the NLRP3 inflammasome [87] and NF-κB via the TLR2/SIRT1/NF-κB pathway [88,89]. Similarly, in a study on cell cultures in dogs, RES-mediated activation of SIRT1 contributed to significant inhibition of NF-κB activation while suppressing the phosphorylation and degradation of IκBa, which is responsible for inhibiting NF-κB [90]. The same study showed that SIRT1 can interact with p300 and form a SIRT1-p300 complex with it, thereby reducing the efficiency of the RANKL-p300-NF-κB pathway while disrupting the interaction between NF-κB and the p65 subunit [90]. p65 is recognized as one of the major activating factors of NF-κB.

In vitro, it was shown that fibroblast cells synthesized more IL-1β, IL-6, and TNF-α mRNA when p65 expression was increased [91]. The pro-inflammatory effects of NF-κB have been detected in cats, so data on the suppression of this pathway by SIRT1 in feline fibroblast cells can be extrapolated to dogs [91]. SIRT1 expression also occurs in canine peripheral cells in lymphocytes, granulocytes, and monocytes, among others [92], suggesting that RES levels may influence leukocyte survival parameters, their activity in cytokine production, and the performance of oxidative bursts. Indeed, one study measured the effect of RES supplementation in dogs on changes in the expression of certain genes in leukocytes [93]. Six genes were significantly different compared with the control group. Genes responsible for neutrophil migration and activation, lymphocyte adhesion, and phagocytes were characterized by reduced expression [93]. By these mechanisms, daily human consumption of RES resulted in reduced levels of CRP and TNF-α and an improved IL-6/IL-10 ratio [94,95]. Interestingly, in vitro studies describing the effects of RES on canine leukocytes indicate an increase in TNF-a and Il-6 production [96], or a decrease in TNF-a in renal epithelial cells [97]. Furthermore, a decrease in il-10 production and an increase in the TNF:il10 ratio in RES-treated cells was discovered [96]. The results obtained contradict the expected effect of RES, and the planned evaluation of the effect of RES in vivo studies in healthy dogs did not yield conclusive results [98]. In a study in dogs, RES increased TNF-a production but did not significantly alter il-6 and il-10 production [98]. The unexpected effect was probably dependent on the dose of RES used in the study.

### 3.2. Resveratrol and Oxidative Stress

As indicated in previous studies, low doses result in improved oxidative status and high doses lead to immunosuppression [99]. Consistent with this, moderate, physiological doses only resulted in reduced oxidative burst and leukocyte stimulation in both in vitro and in vivo studies [96,98]. However, it is not known what effect higher doses of RES would have on the dog’s body, as the first and only NOEL that was determined was 600 mg/kg b.w., in which relatively low doses were tested [46,48]. High doses of RES in experimental studies led to reduced infiltration of inflammatory cells into the endocardial tissue [100]. The effect of RES on the expression of genes related to leukocyte adhesion capacity may explain this effect [93]. In addition, cell migration may be limited by the effect of RES on the cytoskeletal structure and cytoskeletal fiber composition [101]. RES modifies the Rac/PAK/MLC and Rac/WASP/ARP pathways that regulate the epithelial–mesenchymal transition, reducing the migratory capacity of the cell [101]. In addition, high doses caused a concomitant decrease in the production of Il-6 and bone morphogenetic protein 2 (BMP-2) [100]. BMP-2 is a regulator of oxidative stress and vascular calcification [102,103], so Il-6 activity is strongly associated with BMP-2. A further component of the BMP-2 activation pathway in the regulation of the inflammatory response is Wnt [103]. In other studies, RES stimulated psi Wnt, promoting cell proliferation and differentiation [104]. The previously mentioned oxidative burst is one of the elements shaping oxidative status, and its presence is necessary for a normal cellular response. RES can promote antioxidant systems and modulate cellular redox state signaling pathways, and its modulating effect on oxidative status is already noticeable at low doses of RES [46,48]. RES, depending on its form, concentration, and associated substances, reduces ROS [105]. Oxidative stress induced in canine lens epithelial cells was reduced by pure RES by about 13.4%, and grape seed and peel extract reduced ROS by 37.1% [106]. In contrast, the established antioxidant, vitamin E, reduced ROS production by 68% [106].

Another study on canine lens epithelial cells demonstrated that the extract may have greater abilities than the n-acetyl cysteine (NAC) [107]. However, it is not clear what the difference in properties is between RES and NAC. Malanodialdehyde, which is one of the markers of oxidative stress, was not altered by RES in dogs, even during long-term use [105]. In contrast, it has been well-established that NAC reduces MAD levels [108]. However, Kukovska et al. described the effect of RES in the case of exercise [105], and in the case of disease states (Trypanosoma brucei infection), lower MAD levels were recorded in the RES-treated group [16].

According to studies, RES is not the only active substance in grapes responsible for suppressing oxidative stress, and RES alone is weaker than the extract [106]. In a study comparing RES with seed and peel extracts, the latter significantly reduced the activity of p38 mitogen-activated protein kinase (MAPK), extracellular signal-regulated kinase 1/2 (ERK ½), stress-activated protein kinase (SAPK)/Jun amino-terminal kinase (JNK) (SAPK/JNK), and serine/threonine kinases (Akt ½) [106,109]. However, the RES-supplemented extract inhibited ERK1/2 and SAPK/JNK better, which may suggest that RES additionally acts via other signaling pathways, or that other components of the extract also have a strong oxido-modulating effect [106]. The main pathway of antioxidant action of RES is the regulation of SIRT1/FOXO [110]. Representatives of the FOXO class, such as FOXO1 and FOXO3A, are important modulators of the cellular response under stress, stimulate antioxidant defense, and regulate the cell cycle [111]. Thus, in the canine, FOXO induction by SIRT1 leads to increased expression of apoptotic and antioxidant proteins [112]. Rat and mouse blood vessel wall cells responded to RES treatment with increased production of SOD and catalase [113,114]. Cultures of cell lines derived from dogs responded with a twofold increased production of SOD-1 [83] and increased production of SOD-2 [97].

However, catalase production was decreased [83]. SOD and catalase, together with γ-GCS, are the three most active antioxidants. In the case of γ-GCS, RES did not increase its production but increased the effectiveness of genistein treatment, so the combination of RES and genistein caused the greatest increase in γ-GCS production [83]. In the same study, RES had an inhibitory effect on the production of pro-oxidant enzymes. NADPH oxidase is one of the main producers of ROS [83,115]. NADPH requires the cooperation of regulatory subunits to function effectively, such as p22phox, where its production is increased by RES administration [83]. The production of another regulator of NADPH activity, p47phox [115], was reduced with RES to 50% of the initial production [83]. The antagonistic action of RES on the two components of this system so far seems little understood, but the very interaction between RES and this fragment of the cell’s redox system is a prospect for further research into its utility for the cardiovascular system, as the associations between NADPH and p22 and p47 play a key role in cardiac remodeling [116]. A separate RES target in the regulation of oxidative stress is nuclear erythroid factor 2-related factor 2 (Nrf2) [117,118]. The activation of NrF2 is mediated by mitogen-activated protein kinases (MAPKs). Among the MAPKs activated by RES, p38, ERK ½ can be distinguished [88]. In the latter-mentioned pathway, NrF2 activates the transcription of antioxidant enzymes [119]. RES supplementation alleviates acute inflammation in dogs by inhibiting NF-κB and simultaneously activating NrF2 [120]. Reduced expression of p38 kinase (one of the MAPK family members) results in increased expression of genes associated with NrF2 activity [121]. despite the expected inhibition of p38 activity after RES administration, did not occur in one study, and even P38 production was increased [106]. However, the p38 kinase, like the SAPK/JNK pathway, is activated by various cellular stresses, including inflammatory cytokines, and is therefore related to the body’s immune response. In addition, activation of p38 MAPK could induce feedback that leads to activation of the NrF2 signaling pathway [122].

Long-term oxidative distress leads to the accumulation of ROS and DNA damage by ROS, inducing the activation of apoptosis pathways, among others, through the SIRT1/FOXO pathway, but also through the activation of the p53 protein [123,124]. Although the p53 protein is mainly identified with inhibitory effects on tumor growth, p53 activity is also anti-inflammatory [125]. This is consistent with the key anti-tumor effect of p53, as it inhibits inflammatory sites that are predisposed to carcinogenesis [126]. Inflammatory effects are reduced by inhibiting APE1/Ref1, which is responsible for the development of inflammation and ROS production [127]. The protective effect of SIRT1 through p53 activation is illustrated by p53 deficiency. It triggers systemic inflammation through WNT activation [126]. There are probably many interactions along the SIRT1-p53 lineage due to their common involvement in aging processes and anti-inflammatory effects in both humans and dogs, which are unfortunately still not well understood [86,128]. For example, the p53 protein is also induced by excessively short telomeres, leading to the activation of apoptotic pathways [129]. At the same time, SIRT1 delays aging by inhibiting telomere attrition, maintaining genome integrity, and promoting DNA damage repair [130]. On average, shorter telomere lengths are associated with death from cardiovascular disease in dogs as well as humans [131,132,133]. A critical enzyme whose activity can slow telomere shortening is telomerase. As indicated by researchers, RES increases telomerase activation, which is essential for maintaining telomere integrity and genome stability in heart cells [134]. Telomerase activity was also increased in the canine adipose tissue cells of a 7-year-old beagle dog by RES [135]. Moreover, in this case, the dose of RES was crucial for the expected effect, as telomerase activity was shown to be highest in samples with the highest concentration of RES (25 μM RES) [135].

## 4. Effects of Resveratrol on Mitochondria

Excessive amounts of ROS are formed because of oxygen metabolism disorders. The main site of redox reactions is the mitochondria, and their key role in heart health and disease is also undeniable. They regulate the energetic and oxidative homeostasis of cardiac tissue cells, the blood vessel wall, and circulating morphotic elements in the body, and mitochondrial efficiency is crucial to cardiac muscle function [136]. Measuring the rate of cellular oxygen consumption (OCR) is essential to assess the role of mitochondria in physiology and pathophysiology. An increase in OCR is directly related to the activity of the mitochondrial respiratory chain complexes and mitochondrial respirasomes [137]. OCR decreases, for example, under general anesthesia, when the cardiac minute volume is lower and oxygen circulation is limited [138]. However, OCR may be reduced in cardiac patients [139]. RES administration to dogs was shown to increase basal OCR and maximal respiration in large-breed pups [140]. The increase in ATP/AMP levels upon cellular stress activates AMPK, inhibiting complex I in mitochondria [141]. RES has been shown to have the ability to improve the function of complex I in mitochondria [141], and under the influence of RES supplementation, glucose metabolism is also altered in small- and large-breed dogs [140]. These changes were accompanied by an increase in pyruvate dehydrogenase (PDH) activity [142]. The progression of heart disease is also associated with worsening cardiomyocyte damage and the activation of apoptotic pathways. Cell cycle and apoptosis pathways are controlled by the mitochondrial membrane protein BCL-2. BCL-2 levels were found to increase under RES in myocardial ischemia–reperfusion injury [143], and this increase was accompanied by a reduction in cardiomyocyte damage. A similar result was obtained when RES was used as a canine semen supplement [144]. Supplementation with 200 μM RES also contributed to a decrease in the level of *OGG1*, the gene responsible for the removal of 8-oxo-guanine, one of the by-products of ROS exposure [144]. At the same time, no change was observed in the mitochondrial membrane protein ROMO1, which is responsible for increasing ROS concentrations [144]. This suggests that RES may have increased the antioxidant response (mechanisms from Section 3). The effect of RES on mitochondria is explained by the effect of RES on SIRT1, which plays a key role in regulating the redox state and energy metabolism. Increased expression of SIRT1 also results in increased activity of complex I [145]. RES supplementation with a SIRT1 inhibitor did not increase bcl-2 protein, and ischemia–reperfusion injury led to worsened cardiomyocyte injury [143]. In addition, a decrease in the proapoptotic protein BAX was noted. With RES sperm supplementation, BAX protein was also decreased compared with the control sample [144]. In conclusion, RES may protect mitochondrial and cardiomyocyte function by decreasing apoptotic pathways.

## 5. Cardioprotective Activity of Resveratrol in Humans

Some recent papers provide an excellent summary of the impact of RES on the cardiovascular disease landscape [11,65,146]. To explore the utility of RES, a number of papers have documented its effect on CVD in preclinical studies in mouse and rat models [11,147,148], as well as in clinical trials [65,149]. The disease entities for which the utility of RES has been detected are conditions originating from blood flow disorders. Circulatory changes in animal models were induced mainly by obliteration of the main supply vessels. A leading example is mouse models of myocardial infarction induced by left coronary artery ligation [150]. At a sufficiently high dose, RES supplementation reduced infarct size and improved survival outcomes in mice [151,152]. Different results were observed at lower doses, varying from no effect on any of the parameters [151] to studies showing a reduction in infarct size [153]. However, dose effects have also been documented in rats, among which only the lowest dose (5 mg/kg/day) showed no effect on survival [154]. To date, there is a lack of treatment data demonstrating the effect of resveratrol in reducing infarct size in humans. More data are available on the ischemic conditions that can lead to myocardial infarction and the effect of RES on these disorders. In the blood of patients suffering from acute coronary disease who received RES, a decrease in inflammatory markers was noted, which was also associated with a decrease in lipid oxidation products [155]. Patients with atherosclerosis as a disease with a potential inflammatory background [156,157], regulated by inflammatory cells [158,159], may benefit from RES supplementation because of its biological activities. Damage to the endothelium of blood vessels becomes a site for the accumulation of cholesterol oxidation products, with consequent stiffening of the vasculature and a reduction in its lumen [160,161]. Using a high-fat diet in Yorkshire swine can induce a disease phenotypically similar to human atherosclerosis [162]. RES supplementation in this model improved cardiac function and induced VEGF-mediated repair processes [163]. In humans, on the other hand, several studies have detected a cholesterol-lowering effect [164] and antioxidant activity [95]. These results, however, are in contrast to the far greater number of studies in which the impact of RES was not detected [165,166]. Other positive aspects of the use of RES are the reduction in blood pressure—e.g., in hypertension [164] and anti-hypertrophic effects on the myocardium [167]. Unfortunately, only a subset of papers show promising results from the use of RES in patients with hypertension [11]. A study conducted in 2016 indicated that high blood pressure was controlled and reduced to reference levels [168]. In a further study, less satisfactory results only indicated reductions in diastolic pressure with a higher dose of RES (300 mg/day) [169]. In another study, a reduction in systolic blood pressure was observed by using RES at a dose of ≥150 mg/day [170].

## 6. Cardioprotective Activity of Resveratrol in Dogs

There is a distinct absence of studies in veterinary medicine similar to those on CVD and RES in humans. However, given the similarities previously discussed, it is conceivable that the benefits of RES could be correlated with those obtained in humans. We divided the cardioprotective effects into subsections because of the multiple biological activities of RES, each mechanism of which may act directly or indirectly on canine cardiovascular function and health.

### 6.1. Antiplatelet Action

One of the first appreciated properties of RES in the context of CVD was its antiplatelet and anticoagulant effects [23,171]. Excessive or abnormal platelet aggregation in dogs can lead to thromboembolic events such as myocardial ischemia, stroke, or acute limb ischemia [172,173,174]. Although dogs, besides sheep, are most similar to humans in terms of their coagulation system, antiplatelet effects may differ from those reported in humans because of species differences [175]. For example, marked differences have been shown between dogs and humans in terms of external activation of coagulation [176]. Despite the differences, RES in individual forms has also shown antiplatelet activity in dogs [177,178]. The key to effective antiplatelet therapy is to influence Virchow’s triad, the three main mechanisms of pathological thrombus formation [179]. The ideal preparation should increase blood flow by removing any stasis, counteract hypercoagulability, and support vascular endothelial function. In the following subsections, the effect of RES on the different elements of Virchow’s triad is presented.

#### 6.1.1. Blood Flow

**Figure 2:**
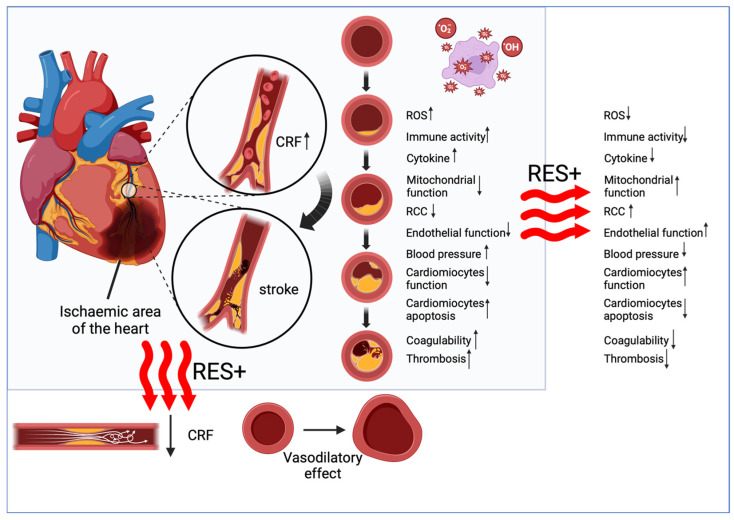
Impact of the resveratrol on cardiovascular health. CRF—cyclic reduction flow; RCC—Cellular Redox Capacity.

Ischemic heart disease, which can result from reduced flow through the coronary arteries, also has its origin in abnormal platelet activation (Figure 2). The occurrence of coronary embolism is the consequence of a decrease in the vessel lumen. The health consequences can be counteracted with antiplatelet preparations that improve flow [180]. Severe myocardial ischemia is much more common in human medicine, but experimental [181] and clinical [182] cases of ischemia are reported in veterinary medicine. The difference is that the main cause of ischemic heart disease in humans is atherosclerosis, which leads to a reduction in the lumen of the coronary arteries [183,184]. In dogs, on the other hand, intimal atherosclerotic vascular degenerative changes are rare and are most often associated with severe thyroid insufficiency [182]. No less, segmental coronary stenosis or malformations in dogs, for example, occur as structural abnormalities [185]. It is thought that RES may have a protective effect against the development of coronary artery disease in humans [186]. In an experimental coronary artery stenosis in dogs, which was designed to mimic an atherosclerotic narrowing of the arterial lumen, the effect of intravenous and gastric administration of red wine was shown to reduce cyclic flow reduction (CFR) [20]. Improvements in coronary flow have also been associated with the effects of alcohol, which may have antiplatelet activity, so additional trials have been designed [23,187,188]. Reductions in CFR were also seen in trials with grape juice alone, but the effect in these trials (without alcohol) was seen 10 min later [23]. In contrast, other products, such as grapefruit juice, containing much lower concentrations of RES did not present similar properties to grape juice [189]. The dose-dependent effects of RES on the cardiovascular system of dogs are also confirmed by studies on the use of grape skin and grape seeds [178]. Using them separately resulted in a weaker antiplatelet effect than giving them to dogs together [178]. Increased vascular flow may also have been produced by the effect of RES on the coronary arteries. In vitro studies showed that the wall of canine blood vessels exposed to direct RES indicated vasodilatory effects [190]. More specifically, RES decreased the sensitivity and maximal contractile response of vascular muscle strips [190].

According to the study, RES may have a potential positive antiarrhythmic effect, thereby increasing cardiac efficiency and consequently reducing thrombus formation. The development of atrial fibrillation (AF) occurs through several signaling pathways and involves structural and electrical remodeling [191]. Several antioxidant effects, as well as inhibition of flow through ion channels [192,193], make RES a promising agent against AF. In studies where low doses of RES were tested, no effect on canine ECG or blood pressure was detected in healthy dogs, and a strong cardioprotective effect was obtained in rats, in which RES attenuated the level of hypertrophy due to tachycardia [46]. In a canine model of atrial fibrillation, the effect of RES (C1)-based drug administration on the length of the effective refractory period (AERP) was observed [192]. In the arrhythmia that was produced, an AERP of less than 80 ms was obtained, while C1 administration increased its time to approximately 87 ms. In addition, a significantly lower total AF duration per day was obtained [192], and C1 did not affect the length of the QT interval and thus did not increase the possibility of ventricular arrhythmias [192]. AF in humans is the most common cause of thromboembolic events. AF is also the most common arrhythmia in humans and dogs [8,194]. However, intracardiac thrombus formation in dogs is less common than in humans and is unlikely to be associated with AF [17,195,196]. Similarly, for distal vascular emboli, AF plays a much smaller role in thrombus formation in dogs compared with cats and humans [197]. In a study looking at cases of aortic thrombus, no structural heart disease was found in dogs [21]. In another study, only a proportion of dogs had cardiovascular disease [197]. In contrast, the consensus of the American College of Veterinary Emergency and Critical Care (ACVECC) denies the influence of cardiac disease in increasing the risk of thrombus in dogs [17].

#### 6.1.2. Hypercoagulability

If thrombus formation in dogs is not as closely linked to blood flow as it is in humans, then RES could act by inhibiting coagulation activators. RES has been shown to reduce platelet aggregation [12], even in individuals who have developed aspirin resistance [198]. The activity is due to inhibited COX-1, reduced thromboxane production, and Ca^2+^ ion efflux, which activates platelet aggregation but may also enhance platelet apoptosis [12,199,200]. As previously mentioned, cardiac disease does not increase the risk of thrombus formation, but platelet-activating factors such as proinflammatory cytokines and serotonin are upregulated in cardiac disease states [201,202,203,204]. There also appears to be an interesting thesis of in situ thrombus formation in the abdominal aorta, in which progressive embolization leads to limb ischemia in dogs [21]. Hypercoagulability may explain this [205]. Hypercoagulability has additionally been linked to the occurrence of pulmonary thrombosis in dogs [206]. The risk factors associated with the development of aortic thrombosis have still not been identified, and treatment is mainly based on the administration of heparin [21]. Perhaps the properties of RES, which reduces overall blood calcium ion levels in dogs, would be useful [100].

#### 6.1.3. Endothelial Dysfunction

The vascular endothelium, the inner layer of the vessel, is directly exposed to pressure changes in the bloodstream. Individual fluctuations in blood flow can trigger the strong secretory activity of normally modestly secreted inflammatory factors. Endothelial dysfunction may be the primary lesion that results in chronic inflammation, accompanied by a loss of anticoagulant factors and an increase in vasoconstrictor and prothrombotic products, as well as abnormal vascular reactivity, which increases the risk of CVD events [207]. Damage to endothelial function also has a cardiogenic basis—a stressful effect is exerted on the vessel wall through altered vascular perfusion values [202]. The most common is venous pulmonary hypertension (PVH), which develops as a result of left ventricular disease (e.g., mitral valve regurgitation) [208]. In humans, pulmonary hypertension is much more often caused by pulmonary arterial hypertension (PAH), which is a primary vasculopathy, than in dogs. In PAH, pressure reduction under RES has been demonstrated in rat and mouse models [164].

Similar factors are involved in mediating the disease process of PAH and PVH, disrupting normal vascular tone. Endothelial dysfunction leads to impaired vasoconstriction and vasodilatation processes, i.e., impaired metabolism of endothelin (ET), serotonin, and nitric oxide, among others [209]. The accumulation of ET is caused by reduced pulmonary clearance due to impaired vascular perfusion, while oxidative stress and mitochondrial dysfunction lead to an impaired release of platelet-accumulated serotonin. In addition, the main vasodilator, NO, decreases its activity due to dysfunction of the endothelium, which is the site of NO production, because of increased vascular pressure, among other factors [210]. Pulmonary vasodilators (such as sildenafil, or prostacyclin derivatives such as beraprost sodium) are key in treatment to promote flow. In addition, antioxidant agents that protect against cardiac and vascular tissue remodeling and support vascular endothelial function appear to be useful [211]. Endothelial dysfunction in dogs has been assessed by levels of C-reactive protein (CRP), nitrate and nitrite (NOx), l-arginine (l-Arg), asymmetric dimethylarginine (ADMA), symmetric dimethylarginine (SDMA), and the von Willebrand factor (vWF) [202]. Increased activity of inflammatory factors and deteriorating endothelial function correlated with cardiac disease status. The effect of RES detected in humans may be twofold. Firstly, it promotes endothelial function by increasing endothelial NOS (eNOS) expression [212]. In addition, the antioxidant action of RES abolishes the adverse effects of ROS on the vascular endothelium [213]. The reduction in ROS and cellular oxidative activity in dogs was described in Section 3. Increased NO production contributes to the recovery of vasodilatation capacity [214]. One study showed that the rat aortic wall exposed to RES underwent dilatation [215]. Similar results were obtained in dogs, where the vasoconstrictor response was reduced [190]. In the rat aorta, this response was mediated by a decrease in PI3K activity and also in the PI3K/Akt pathway [215]. Moreover, RES restored SIRT1 synthesis, which may contribute to the post-translational deacetylation of eNOS lysine residues [216]. Endothelial function is also directly related to mitochondrial function, as described in Section 4. RES in dogs can stimulate similar mitochondrial and cellular pathways controlling oxidative stress, so, presumably, the restoration of eNOS and vascular function is mediated by analogous pathways as in rodents and humans. However, studies on the effects of RES on systolic and diastolic blood pressure values in dogs are still not available.

### 6.2. Cancers and Resveratrol

Cardio-oncological management has been compared to a double-edged sword because of its very good therapeutic effects; however, the therapy is extremely toxic to the cardiovascular system [217]. Few papers address the effects of RES on canine cardiovascular disease. It does, however, increase cellular oxidative capacity, thus having the potential to silence inflammatory foci predisposed to carcinogenesis and increase myocardial energy yield [140]. In addition, a reduction in DNA damage has been shown following RES treatment in dogs (as part of cancer therapy) [218]. Hemangiosarcoma cells, a tumor of vascular endothelium as well as cardiac tissue, have been studied twice [219,220,221]. RES promoted the pro-apoptotic AND tumor growth inhibitory effects of doxorubicin in the treatment of hemangiosarcoma [220]. Perhaps RES facilitates doxorubicin target site attainment by improving vascular endothelial function and abolishing oxidative stress. Doxorubicin, which is used in the treatment of hemangiosarcoma, does not need to cross barriers such as the blood–brain barrier or the blood–nucleus barrier, but RES could hinder the therapeutic effect through its effect on P-glycoprotein [222]. The impairment of its function leads to a decrease in the efficiency of transmembrane transport mechanisms, including for drugs, and an increase in the penetration capacity of drugs [223,224]. Canine MDCKII cells treated with RES achieved a significantly more efficient p-glycoprotein-mediated efflux effect [225]. However, this could translate into reduced cardiotoxicity of cancer drugs by nullifying cellular accumulation. A stand-alone antiproliferative effect of RES or its two oligomers, hopeaphenol and r2-viniferin, has also been demonstrated in histiocytic sarcoma [221]. It has been indicated that the supportive effects of RES may have similar mechanisms to those in humans. Effects on the p38 MAPK, AMPK, and ERK1/2 pathways have been demonstrated [220]. The use of RES in oncology may be hampered by its effects on key cell differentiation pathways, thereby promoting the differentiation of some cancers [109]. RES induced melanoma cell differentiation via the c-Jun N-terminal kinase (JNK) inhibitor pathway [109]. Similarly, inhibitory effects on this signaling pathway have also been demonstrated on non-cancerous cells [106].

### 6.3. Usage of Resveratrol during Surgery

Dogs undergo a variety of surgical procedures to aid heart function. In human medicine, the properties of RES have recently been recognized as a potentially good agent for influencing the healing of facial bone fractures, which could find application in the recovery period [226]. In dogs, atrioventricular valve repair requires antiplatelet treatment. Veterinary medicine has well-functioning antiplatelet therapy (aspirin, clopidogrel) [227] and anticoagulation therapies (heparin, warfarin) [228]. However, as reported by human medics, there are complications associated with the inflammatory response and calcification of biological valves [229,230,231]. In a canine model, RES, through its anti-inflammatory and antioxidant effects, inhibited local infiltration of leukocytes and fibroblasts [100]. In particular, inhibition of basophils, which are found in significant numbers at sites of calcification and may be particularly important for calcification, appeared to be important [232]. Early calcium deposition is evident because of a permeable substance in the extracellular matrix that results from basophil activity [100]. In the control group, such changes were present in the peri-implant tissue, which was not demonstrated in the RES-treated group [100]. Furthermore, it was discovered that RES may have a protective effect against the development of ischemia–reperfusion injury [233]. The 7-day administration of RES resulted in greater pressure tolerance to hemorrhage, so RES could be recommended for patients with low pressure in the preoperative period [233]. Other reports indicate a protective effect against hemorrhagic shock and a mitigating effect on glomerular damage due to hemorrhage [234].

## 7. Conclusions and Future Perspectives

Resveratrol has activity in many areas relevant to CVD in dogs and is consistent with effects obtained in rodents and humans. Key antioxidant and anti-inflammatory signaling pathways are activated depending on the dose, form of resveratrol administration, and concomitant substances. Resveratrol is also proving to be a valuable antiplatelet drug that can be used in cardiac patients or patients who qualify for surgery. The improvement in vascular endothelial function, which directly contributes to this, appears to work similarly in humans. The information gathered so far provides a solid basis for further targeted research, which we suggest based on this review. Information on the effects of RES on cardiomyocyte function is lacking in the current state of knowledge. Mouse and rat cardiomyocytes are sensitive to RES, which may protect them from excessive oxidative stress, excessive hypertrophy, apoptosis, and fibrosis of cardiac tissue [235]. In addition, it may positively influence mitochondrial biogenesis or improve myocardial blood supply [235]. As indicated in dogs, SIRT1 mediates cardiac remodeling. Aldosterone, which is an important factor in cardiac fibrosis, inhibits SIRT1-AMPK expression [236]. Therefore, SIRT1 agonists such as RES may be useful for treatment. The current information obtained from in vitro and in vivo studies should be extended to clinical trials. In particular, the pathways responsible for antiplatelet and anticoagulant effects should be tested on a wider group of patients. In particular, we would like to highlight the pressing need to initiate clinical trials using resveratrol in cardiac patients. Of particular interest would be studies on aging animal groups, fitting into the conventions of “Dog Aging Project” studies [237]. We suggest developing novel research strategies in which resveratrol will have higher bioavailability targeting dogs.
